# The diagnostic value of platelet-related indicators to CRP ratio in periprosthetic joint infection

**DOI:** 10.3389/fcimb.2026.1871636

**Published:** 2026-08-07

**Authors:** Runshan Duan, Kai Xuan, Yi Jin, Zhen Wang

**Affiliations:** 1 Department of Orthopaedics, Henan Provincial People’s Hospital, Zhengzhou University People’s, Hospital, Zhengzhou, Henan, China; 2 Department of Hospital Infection Management, Henan Provincial People’s Hospital, Zhengzhou, University People’s Hospital, Zhengzhou, Henan, China

**Keywords:** arthroplasty, blood platelets, C-reactive protein, prosthesis-related infections, ROC curve

## Abstract

**Objective:**

To evaluate the diagnostic value of platelet-to-CRP ratios for periprosthetic joint infection (PJI) following total joint arthroplasty, and to assess the influence of potential confounders.

**Methods:**

A retrospective case-control study included 151 PJI patients and 178 with aseptic loosening (AL). CRP, ESR, PLT, PDW, PLT/CRP, PLT/ESR, and PDW/CRP were compared between groups. ROC curves assessed diagnostic performance. Subgroup analyses stratified by joint (hip/knee) and infection duration (acute/chronic) were performed. Multivariate regression adjusted for time-related covariates, with pairwise ROC comparisons via DeLong test.

**Results:**

Joint distribution, symptom duration, diabetes, and time from arthroplasty differed significantly between groups (all *P* < 0.05); sex, age, laterality, revision history, and immunosuppression did not. All seven biomarkers differed significantly between PJI and AL groups (all *P* < 0.0001). PDW/CRP achieved the highest AUC (0.9047), with 84.3% sensitivity and 84.8% specificity, followed by CRP (AUC = 0.9002) and PLT/CRP (AUC = 0.8841). Single platelet markers performed poorly (AUCs < 0.66). No significant joint-specific differences were observed (all *P* > 0.05). PDW/CRP showed higher AUC in acute (0.9452) versus chronic PJI (0.8901, *P* = 0.0407). All seven biomarkers remained significantly associated with PJI after adjustment for time-related variables (all *P* < 0.01). DeLong tests confirmed that PLT/CRP and PDW/CRP were comparable to CRP alone (both *P* > 0.05), while significantly outperforming single platelet parameters (all *P* < 0.001).

**Conclusion:**

PLT/CRP and PDW/CRP are low-cost adjunctive biomarkers for PJI diagnosis, with PDW/CRP offering well-balanced sensitivity and specificity. Their diagnostic performance is stable across hip and knee joints and independent of symptom duration or implantation time. External validation in unselected populations is warranted.

## Introduction

Total joint arthroplasty is one of the most commonly used methods for treating end-stage joint diseases to alleviate pain and disability (Compagnoni et al., 2018; Gholson et al., 2018; Hamilton et al., 2018). It is estimated that the demand for joint replacement surgery is expected to increase significantly in the future due to the extension of life expectancy and the substantial costs of health and social care caused by osteoarthritis and other age-related joint diseases (Singh et al., 2019; Rupp et al., 2020). Although joint arthroplasty surgery is safe, complications still exist. Infection after artificial joint replacement is one of the most destructive complications after total joint arthroplasty, imposing a huge burden on patients, surgeons and the healthcare system. The incidence of periprosthetic joint infection (PJI) varies among different centers, but it is mainly concentrated between 0.5% and 1.0% in the hip joint and between 0.5% and 2% in knee arthroplasty (Nair et al., 2017; Beam and Osmon, 2018). Although the incidence of infection around joint prostheses is very low, the consequences of PJI can be catastrophic, potentially leading to prosthesis failure, revision surgery, and even amputation. Early and accurate diagnosis is crucial for improving prognosis. However, the clinical manifestations of PJI are diverse, lacking specific symptoms, and traditional diagnostic methods have limitations. At present, the internationally recognized diagnostic criteria for PJI mainly rely on serum inflammatory markers (such as CRP, ESR), joint fluid analysis, microbial culture and histopathological examination (Parvizi et al., 2011; Parvizi et al., 2014). However, the sensitivity or specificity of a single indicator is insufficient. Although combined detection can improve accuracy, it increases medical costs and time consumption.

C-Reactive protein (CRP) holds significant clinical value in the diagnosis and monitoring of PJI. It is synthesized in the liver and adipose tissue within 6 hours after bacterial infection, inflammatory disease or tissue damage, and is one of the core inflammatory markers for the assessment of infection after current joint replacement surgery. The sensitivity of CRP in acute PJI can reach over 90%, but its specificity is relatively low (about 70%-80%), as early postoperative and non-infectious inflammation (such as deep vein thrombosis and hematoma) can also lead to an increase in CRP. Research shows that CRP combined with erythrocyte sedimentation rate (ESR) can significantly improve diagnostic accuracy. Both are core serological indicators of the PJI diagnostic criteria for MSIS (Musculoskeletal Infection Society) (Parvizi et al., 2018) in 2018 and ICM (International Consensus Conference) (Osmon et al., 2013) in 2013. However, it should be noted that some low-virulence infections (such as coagulase-negative staphylococcus) may only present with a mild increase or normal CRP.

The research on platelets and their related indicators in the diagnosis of inflammation and infectious diseases has been a hot topic in recent years (Guclu and Faruq Agan, 2017; Demirdal and Sen, 2018; Paziuk et al., 2020; Tirumala et al., 2021; Sun et al., 2025). Under infection, inflammatory factors stimulate the increased production of bone marrow megakaryocytes, leading to elevated platelet reactivity. Meanwhile, platelets participate in inflammatory reactions and aggregate at the site of infection as immune cells. Studies have shown that the morphological indicators of platelets, including platelet distribution width (PDW) and mean platelet volume (MPV), are affected by infection. However, existing evidence indicates that their diagnostic value alone is limited (Song et al., 2024), and they are mainly used as auxiliary indicators in combination with other inflammatory markers. Multiple studies have shown that platelets have certain reference value in determining the diagnosis of periprosthetic joint infection, but the diagnostic efficacy varies (Song et al., 2024) (Paziuk et al., 2020), which may be related to individual differences, underlying diseases, and other factors.

To date, only a limited number of studies have evaluated composite hematological indices for the diagnosis of periprosthetic joint infection, and a universal consensus is still lacking. A retrospective cohort study found that the neutrophil-to-lymphocyte ratio (NLR), monocyte-to-lymphocyte ratio (MLR), and platelet-to-lymphocyte ratio (PLR) were all significantly elevated in patients with PJI relative to those with aseptic failure. Recent studies have highlighted that CRP needs to be combined with other markers to improve diagnostic efficiency (Christopher et al., 2021; Yigit et al., 2021; Dayan et al., 2026). The platelet-to-C-reactive protein (PLT/CRP) is the ratio of platelet count to CRP concentration, which theoretically can simultaneously reflect the degree of inflammatory activation and the immune status of the body. Previous studies have reported that CRP/PLT is valuable in the diagnosis of diseases such as sepsis (Li et al., 2021; Chen et al., 2026), but its application research in the field of PJI is still scarce. PDW is a parameter in blood routine tests, reflecting the degree of difference in platelet volume size (i.e., platelet heterogeneity). Under inflammatory conditions such as bacterial infections, platelets are activated and participated in immune responses, leading to changes in PDW. However, research on PDW in PJI is still relatively scarce, and the application of a single PDW indicator is limited. It is necessary to combine it with other indicators for the diagnosis of infection. Therefore, this study aims to explore the clinical value of the ratio of PLT and its related indicators to CRP in the diagnosis of PJI, and to compare it with traditional single inflammatory indicators, providing a new reference basis for the early diagnosis of PJI.

## Materials and methods

A retrospective case-control study was conducted to collect the clinical data of patients with PJI and aseptic loosening who underwent revision and were admitted to the orthopedics department of our hospital from January 2017 to December 2025. Inclusion criteria:①Infection or loosening manifestations such as joint pain, swelling, and dysfunction occur after the initial or revised artificial joint replacement surgery; ②The clinical data are complete, including preoperative serological tests, microbiological cultures. ③Age≥18 years old. Exclusion criteria: ①Concurrent active infectious diseases (such as respiratory tract infections, urinary tract infections, etc.); Administration of antibiotics prior to admission or blood sampling. ②Combined with hematological diseases. ③Clinicians make a comprehensive judgment based on the patient’s clinical manifestations and serological/microbiological cultures, to rule out suspected PJI infection patients. ④Patients with incomplete medical records and test data; ⑤Before admission, a joint cement spacer had been implanted. This retrospective study was conducted in accordance with the principles of the Declaration of Helsinki. The study used anonymized clinical data, and the requirement for informed consent was waived by the Medical Ethics Committee of Henan Provincial People’s Hospital due to the retrospective nature of the research and the use of de-identified patient data.

The diagnosis of periprosthetic joint infection (PJI) was established in accordance with the 2018 Musculoskeletal Infection Society (MSIS) criteria and the 2013 International Consensus Meeting (ICM) guidelines.

The AL group was defined as patients with progressive radiolucent lines (>2 mm) or component migration on serial radiographs, without clinical signs of infection (e.g., sinus tract, fever, or persistent pain), and with intraoperative findings of no purulence. All AL patients underwent at least two intraoperative tissue cultures and histopathological examination. Cases with positive cultures or suspicious histopathology were excluded. For low-virulence organisms (e.g., Coagulase-negative staphylococci, Cutibacterium acnes), we required two positive cultures of the same organism or one positive culture with corresponding histopathological inflammation to rule out contamination.

### Study population

**Figure 1:**
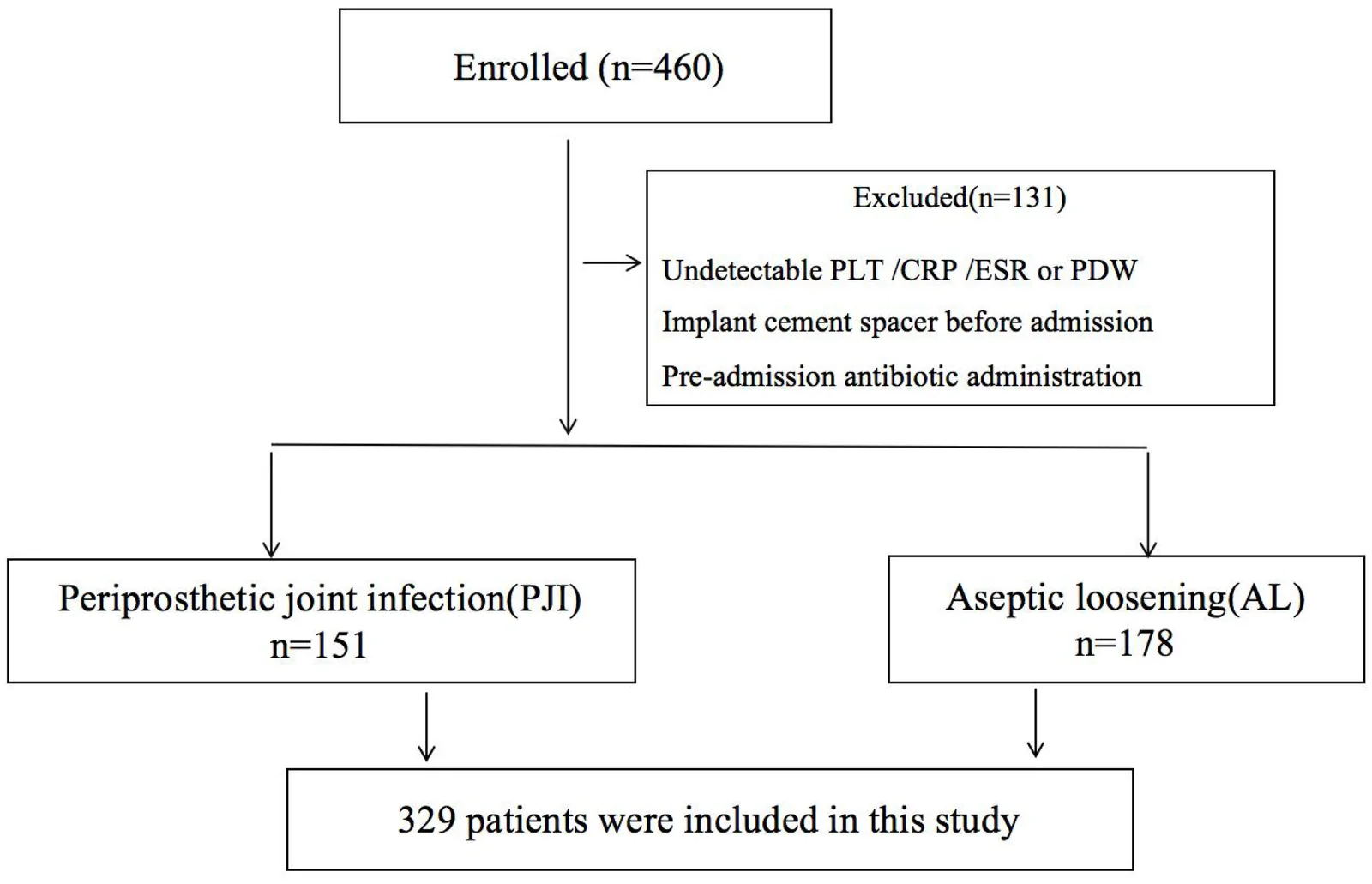
Flow diagram of included patient.

A total of 329 patients were ultimately included and divided into the periprosthetic joint infection group (PJI, 151 cases) and the aseptic loosening group (AL, 178 cases) according to the PJI diagnostic criteria of the International Musculoskeletal Infection Association (MSIS) in 2018 (**Figure 1**).

### Detection methods

Platelet count (PLT) and platelet distribution width (PDW) were detected by a fully automatic blood cell analyzer. C-reactive protein (CRP) was detected by immunoturbidimetry, and erythrocyte sedimentation rate (ESR) was detected by the Westergren method. PLT/CRP is calculated as the ratio of PLT (×10^9^/L) to CRP (mg/L). PLT/ESR is calculated as the ratio of PLT (×10^9^/L) to ESR (mm/h). PDW/CRP is calculated as the ratio of PDW (fL) to CRP (mg/L). All detection operations were carried out strictly in accordance with the reagent instructions and instrument operation procedures. The result data of CRP, ESR, PLT and PDW of patients meeting the inclusion criteria were collected and recorded through the electronic medical record system of our hospital, and the ratios of PLT/CRP, PLT/ESR and PDW/CRP were calculated according to the formulas.

### Statistical methods

Data analysis was performed using GraphPad Prism 7.0a software(GraphPad Software Inc., San Diego, California, USA). The Shapiro-Wilk test was applied to test the normal distribution of continuous measurement data. Normally distributed data are expressed as mean ± standard deviation (x̄ ± s), and between-group comparisons were performed using independent samples t-test. Non-normally distributed data are expressed as median (interquartile range, IQR), and between-group comparisons were conducted using the Mann–Whitney U test. Homogeneity of variance was tested prior to intergroup comparisons; for data with unequal variance, Welch’s t-test was used for normally distributed variables. Categorical data were expressed as n/(%), and χ² tests were used for inter-group comparisons. Receiver Operating Characteristic (ROC) curves were plotted to evaluate the diagnostic performance of each indicator, and the area under the curve (AUC), sensitivity, specificity, and optimal cutoff value were calculated. The optimal cut-off values of all indicators were determined by maximizing Youden’s index (Youden’s index = sensitivity + specificity − 1). At the optimal cut-off value defined by maximum Youden’s index, positive likelihood ratio (LR+) and negative likelihood ratio (LR−) were calculated. LR+ was defined as sensitivity divided by (1 − specificity), and LR− was defined as (1 − sensitivity) divided by specificity. Positive predictive value (PPV) and negative predictive value (NPV) were calculated at the optimal cut-off value of each indicator. PPV was defined as the proportion of true positives among all positive test results, and NPV was defined as the proportion of true negatives among all negative test results.

The ROC results of the detection indicators in the diagnosis of PJI were compared pairwise using the method of Delong et al. (1988) with MedCalc 22.001 statistical software(MedCalc Software, Ostend, Belgium). Subgroup ROC analyses stratified by joint location (hip vs. knee) and infection duration (acute vs. chronic, ≤3 months vs. >3 months) were also conducted, with between-subgroup AUC comparisons performed using the DeLong test.

To ensure that observed differences were attributable to PJI status rather than temporal variations in inflammation, all biomarker analyses were performed using multiple linear regression models adjusted for days since symptom onset and months since implantation. All biomarkers were natural log-transformed (Ln[Y + 1]) prior to regression analysis to improve normality. Biomarkers that remained significantly associated with PJI status after adjustment for time covariates were considered robust. *P* < 0.05 was considered statistically significant.

## Results

**General information of participants**.

A total of 151 PJI patients and 178 aseptic loosening (AL) patients were included in this study. The baseline demographic and clinical characteristics of the two groups are summarized in **Table 1**.There were no significant differences between the PJI and AL groups in terms of gender (*P* = 0.8573), age (*P* = 0.2986), unilateral or bilateral involvement (*P* = 0.248), history of prior revision surgery (*P* = 0.11), and glucocorticoid or immunosuppressive use (*P* = 0.1215), indicating good comparability of these baseline features between the two groups. However, statistically significant differences were observed in several clinical parameters. The proportion of hip arthroplasty was significantly higher in the AL group (71.3%) than in the PJI group (47.0%, *P* < 0.0001). Symptom duration was significantly shorter in the PJI group [median 30 days, IQR (7, 180)] compared with the AL group [median 180 days, IQR (60, 730), *P* < 0.0001]. Additionally, the prevalence of diabetes mellitus was significantly higher in the PJI group (25.2%) than in the AL group (14.0%, *P* = 0.0106). The interval from post-arthroplasty to diagnosis was also significantly shorter in the PJI group.

**Table 1:** Comparison of the general data between the two different groups.

Characteristics	PJI group	AL group	*Z (U)*	*P*
Gender (Man/Female)^#^	60 (39.7)/91 (60.3)	69 (38.8)/109 (61.2)	0.1798	0.8573
Age (years)	69 (61-74)	67 (60.75-72)	12546	0.2986
Joint (Hip/Knee)^#^	71 (47.0)/80 (53.0)	127 (71.3)/51 (28.7)	4.492	<0.0001
Symptom duration (d)	30 (7, 180)	180 (60, 730)	7475	<0.0001
Post-arthroplasty interval (mon)	12 (2, 48)	108 (60, 156)	4192	<0.0001
Unilateral or bilateral involvement^#^	117 (77.5)/34 (22.5)	128 (71.9)/50 (28.1)	1.155	0.248
Prior revision surgery^#^	11 (7.3)	6 (3.4)	1.598	0.11
Diabetes mellitus^#^	38 (25.2)	25 (14.0)	2.555	0.0106
Glucocorticoids or immunosuppressive use^#^	4 (2.6)	1 (0.6)	1.549	0.1215

PJI, periprosthetic joint infection; AL, aseptic loosening; IQR, interquartile range. d, days; mo, months. ^#^Categorical variables are presented as n (%), and comparisons were performed using chi-square test. Continuous variables are presented as median (IQR).

### Comparison of detection indicators between the PJI and AL groups of patients

The levels of CRP, ESR and PLT in the PJI group were significantly higher than those in the AL group. However, PDW, PLT/CRP, PLT/ESR and PDW/CRP were all significantly lower than those in the AL group (**Table 2**), and the difference was statistically significant (*P* < 0.05).

**Table 2:** Comparison of detection indicators between the PJI and AL groups.

Indicator	PJI group (n=151)	AL group (n=178)	*U*	*P*
CRP (mg/L)	23.78 (8.4 -56.52)	1.41 (0.5-3.93)	2684	<0.0001
ESR (mm/h)	61 (38-85)	18 (10-33)	3978	<0.0001
PLT (×109/L)	271 (209-332)	225 (181-270)	9273	<0.0001
PDW (fL)	11 (9.5-13)	12.5 (10.9-14.1)	9719	<0.0001
PLT/CRP	12.34 (4.93-33.33)	143.9 (59.56-385.4)	3114	<0.0001
PLT/ESR	4.48 (3.26-7.53)	12.03 (7.45-22.98)	4794	<0.0001
PDW/CRP	0.46 (0.20-1.27)	8.98 (3.07-21.56)	2561	<0.0001

PJI, periprosthetic joint infection; AL, aseptic loosening; Notes: Continuous variables are presented as median (IQR).

### ROC curve analysis of each index for diagnosing PJI

Among the traditional inflammatory markers, CRP achieved the highest AUC of 0.9002, with an optimal cut-off value of 5.835 mg/L, corresponding to a sensitivity of 81.5% and a specificity of 85.4%. ESR yielded an AUC of 0.852 with a cut-off of 44.5 mm/h, showing higher sensitivity (88.8%) but relatively lower specificity (69.5%) compared with CRP. For single platelet parameters, both PLT and PDW demonstrated limited diagnostic capacity, with AUC values of 0.655 and 0.6384, respectively. At their optimal cut-offs (260.5 ×10^9^/L for PLT, 11.15fL for PDW), sensitivity and specificity were both below 75%, indicating that isolated platelet indices are insufficient for accurate PJI diagnosis when used alone. Notably, the platelet-derived composite ratios exhibited substantially improved diagnostic performance compared with single platelet markers. PDW/CRP achieved the highest AUC of 0.9047 among all tested indicators, with an optimal cut-off value of 1.718. At this threshold, PDW/CRP demonstrated a nearly perfectly balanced sensitivity (84.3%) and specificity (84.8%), with a difference of only 0.5% between the two parameters. PLT/CRP yielded an AUC of 0.8841 (cut-off = 35.53), maintaining favorable balance between sensitivity (85.4%) and specificity (79.5%). In comparison, PLT/ESR showed an AUC of 0.8216, which was lower than the CRP-based ratios. Regarding likelihood ratios, PDW/CRP presented the highest positive likelihood ratio (LR+ = 5.532) and a low negative likelihood ratio (LR− = 0.1856), which was comparable to CRP (LR+ = 5.591, LR− = 0.217). These prevalence-independent metrics further support the diagnostic value of platelet-CRP composite ratios.

**Figure 2:**
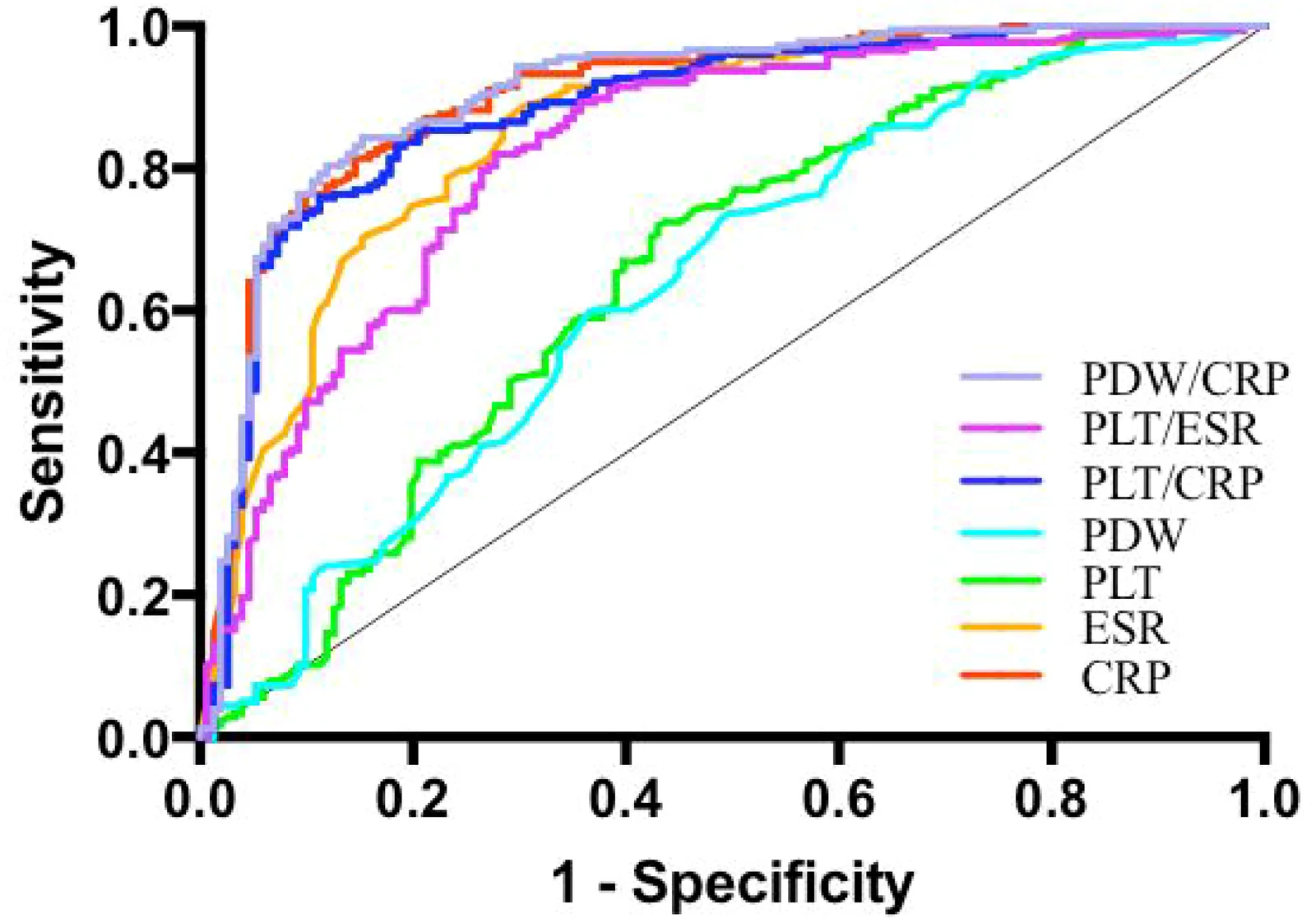
The ROC curves of each detection index for predicting PJI.

In summary, while single platelet markers (PLT, PDW) have limited standalone diagnostic utility, their composite ratios with CRP (especially PDW/CRP) achieve diagnostic performance comparable to CRP alone, with the additional advantage of more balanced sensitivity and specificity. The ROC curve parameters of each indicator are detailed in **Table 3**; **Figure 2**.

**Table 3:** Diagnostic performance of each index in PJI diagnosis.

Indicator	AUC	Optimal cut-off value	Sen	Spe	PPV	NPV	LR+	LR-
CRP (mg/L)	0.9002	5.835	81.5	85.4	79.6	86.8	5.591	0.217
ESR (mm/h)	0.852	44.5	88.8	69.5	84	77.5	2.914	0.1616
PLT (×10^9^/L)	0.655	260.5	71.9	57	63.2	66.3	1.671	0.4932
PDW (fL)	0.6384	11.15	73	51	61.6	63.7	1.49	0.5289
PLT/CRP	0.8841	35.53	85.4	79.5	82.2	83.1	4.159	0.1838
PLT/ESR	0.8216	6.371	82	72.2	77.3	77.7	2.949	0.2491
PDW/CRP	0.9047	1.718	84.3	84.8	82.1	86.7	5.532	0.1856

AUC, area under the curve; Sen, sensitivity; Spe, specificity; PPV, positive predictive value; NPV, negative predictive value; LR+, positive likelihood ratio; LR−, negative likelihood ratio; J, Youden’s index; CRP, C-reactive protein; ESR, erythrocyte sedimentation rate; PLT, platelet count; PDW, platelet distribution width. Note: All values of Sen, Spe, PPV and NPV are expressed as percentages (%).

LR+ values were automatically generated by GraphPad Prism. LR− values were calculated using the formula based on sensitivity and specificity (retained as percentages rounded to two decimal places).

### ROC curve analysis of various indicators for diagnosing PJI in hip and knee joints

To evaluate whether joint location confounds the diagnostic performance of the tested markers, we performed stratified ROC analyses in hip and knee subgroups separately (**Table 4**). Pairwise comparisons of AUCs between hip and knee cohorts were conducted using the DeLong method. No statistically significant difference was observed in the diagnostic efficacy of any marker between hip and knee PJI (all *P* > 0.05), indicating that joint location is not a major confounding factor for these inflammatory indicators. PDW/CRP maintained the highest diagnostic performance in both subgroups, with AUCs of 0.9012 in the hip cohort and 0.9237 in the knee cohort. Notably, PDW/CRP also achieved the most balanced sensitivity and specificity across both joint types: the difference between sensitivity and specificity was only 6.2% for hip PJI and 3.9% for knee PJI. In comparison, CRP showed a larger sensitivity-specificity gap, particularly in the hip subgroup (24.1% difference). PLT/CRP also demonstrated favorable diagnostic performance in both subgroups (hip AUC = 0.8793, knee AUC = 0.9064), while single platelet markers (PLT and PDW) consistently showed poor discriminatory capacity in both hip and knee cohorts, with all AUCs below 0.68. These subgroup findings confirm that platelet-CRP composite ratios, especially PDW/CRP, maintain stable and well-balanced diagnostic performance regardless of joint location.

**Table 4:** ROC curve analysis for diagnosing PJI in hip and knee joints.

Indicator	Hip			Knee			*Z*	*P*
	AUC	Sen	Spe	AUC	Sen	Spe		
CRP	0.8942	71.7	95.8	0.9205	92.2	83.8	0.757	0.4491
ESR	0.842	86.6	71.8	0.8721	98	66.3	0.704	0.4815
PLT	0.6762	73.2	57.8	0.614	70.6	56.3	0.971	0.3317
PDW	0.6489	58.3	67.6	0.6354	72.6	53.8	0.211	0.8329
PLT/CRP	0.8793	73.2	91.6	0.9064	88.2	83.8	0.725	0.4687
PLT/ESR	0.8036	86.6	62	0.852	94.1	72.5	1.035	0.3005
PDW/CRP	0.9012	81.1	87.3	0.9237	90.2	86.3	0.659	0.5101

AUC, area under the curve; Sen, sensitivity; Spe, specificity;CRP,.

C-reactive protein; ESR, erythrocyte sedimentation rate; PLT, platelet count;

PDW, platelet distribution width. Note: All values of Sen and Spe are

expressed as percentages (%).

### ROC Curve Analysis of Various Biomarkers for Diagnosing PJI in Acute and Chronic Joint Infections

To further explore the diagnostic performance of tested markers across different disease stages, we stratified patients into acute PJI (symptom duration ≤ 3 months, n = 40) and chronic PJI (symptom duration > 3 months, n = 111) subgroups and performed ROC analyses (**Table 5**). DeLong tests were used to compare AUCs between the two subgroups.

**Table 5:** ROC curve analysis for diagnosing PJI in acute and chronic joint infections.

Indicator	≤3 months (n=40)			>3 months (n=111)			*Z*	*P*
	AUC	Sen	Spe	AUC	Sen	Spe		
CRP	0.9023	93.3	75	0.8994	81.5	86.5	0.08115	0.9353
ESR	0.7862	70.8	77.5	0.8757	88.8	73.9	-1.776	0.07566
PLT	0.7181	80.9	57.5	0.6323	72.5	54.1	1.364	0.1726
PDW	0.677	86	45	0.6245	59.6	64	0.8482	0.3963
PLT/CRP	0.9114	83.2	85	0.8743	75.8	89.2	1.169	0.2424
PLT/ESR	0.7467	91.6	50	0.8486	82	78.4	-1.999	0.04558
PDW/CRP	0.9452	84.3	92.5	0.8901	80.3	86.5	2.046	0.04071

AUC, area under the curve; Sen, sensitivity; Spe, specificity; CRP,.

C-reactive protein; ESR, erythrocyte sedimentation rate; PLT, platelet count;

PDW, platelet distribution width; PJI, periprosthetic joint infection.

Note: Acute PJI was defined as symptom duration ≤ 3 months, and chronic PJI as > 3

months. All values of Sen and Spe are expressed as percentages (%).

PDW/CRP demonstrated the highest diagnostic accuracy in acute PJI, with an AUC of 0.9452, which was significantly higher than in chronic PJI (AUC = 0.8901, *P* = 0.04071). This finding suggests that PDW/CRP is particularly sensitive to acute inflammatory responses in early-stage PJI. Notably, PDW/CRP also maintained the most balanced sensitivity and specificity in both subgroups, with sensitivity-specificity differences of only 8.2% (acute) and 6.2% (chronic). In contrast, PLT/ESR performed significantly better in chronic PJI (AUC = 0.8486) than in acute PJI (AUC = 0.7467, *P* = 0.04558), consistent with the clinical observation that ESR-related indicators are more sensitive to chronic inflammatory processes. ESR showed a similar trend, with higher AUC in chronic PJI (0.8757 vs. 0.7862), although the difference did not reach statistical significance (P = 0.07566). CRP maintained stable and high diagnostic performance across both acute (AUC = 0.9023) and chronic PJI (AUC = 0.8994), with no significant difference between subgroups (*P* = 0.9353). Single platelet markers (PLT and PDW) showed relatively poor discriminatory capacity in both acute and chronic subgroups, with all AUCs below 0.72. These subgroup results indicate that PDW/CRP has unique advantages in diagnosing acute PJI, while maintaining well-balanced performance across both disease stages.

### ROC curve analysis of various biomarkers for diagnosing PJI caused by *S. aureus* and CoNS

To further evaluate the diagnostic performance across different bacterial pathogens, we performed subgroup ROC analyses in PJI cases caused by Staphylococcus aureus (n = 25) and coagulase-negative staphylococci (CoNS, n = 26) (**Table 6**). DeLong tests were used to compare AUCs between the two subgroups. CRP maintained high diagnostic accuracy in both subgroups, with AUCs of 0.8935 for S. aureus PJI and 0.9325 for CoNS PJI, showing no significant difference (*P* = 0.4604). PDW/CRP also demonstrated stable and favorable diagnostic performance across both pathogen types, with AUCs of 0.8791 and 0.8572 for S. aureus and CoNS, respectively, with no significant between-group difference (*P* = 0.7449). Similarly, PLT/CRP showed comparable diagnostic efficacy in the two subgroups (AUC 0.8604 vs. 0.8388, *P* = 0.7597). Notably, single PLT exhibited significantly different diagnostic performance between the two pathogen types: its AUC was only 0.5147 for S. aureus PJI, indicating nearly no discriminatory capacity, while it reached 0.7356 for CoNS PJI (*P* = 0.0201). However, when combined with CRP as composite ratios, the diagnostic performance became stable across both subgroups, suggesting that the platelet-CRP ratios compensate for the instability of single platelet markers across different pathogen types. PDW showed poor diagnostic efficacy in both subgroups (AUCs < 0.60), with no significant difference between pathogen types (*P* = 0.9574). These findings indicate that platelet-CRP composite ratios, particularly PDW/CRP, maintain stable diagnostic performance regardless of the causative pathogen type, further supporting their clinical utility as robust auxiliary markers.

**Table 6:** ROC curve analysis for diagnosing PJI caused by S. aureus and CoNS.

Indicator	S. aureus (n=25)			CoNS (n=26)			*Z*	*P*
	AUC	Sen	Spe	AUC	Sen	Spe		
CRP	0.8935	93.3	80	0.9325	76.4	100	-0.7382	0.4604
ESR	0.8649	79.8	80	0.8673	70.8	92.3	-0.04038	0.9678
PLT	0.5147	75.3	44	0.7356	67.4	76.9	-2.324	0.02014
PDW	0.5906	89.9	32	0.5957	67.4	53.9	-0.05347	0.9574
PLT/CRP	0.8604	83.7	80	0.8388	85.4	80.8	0.3058	0.7597
PLT/ESR	0.7302	82.6	64	0.801	91.6	61.5	-0.816	0.4145
PDW/CRP	0.8791	86.5	80	0.8572	84.3	80.8	0.3254	0.7449

S. aureus, Staphylococcus aureus; CoNS, coagulase-negative staphylococci;.

AUC, area under the curve; Sen, sensitivity; Spe, specificity; PJI, periprosthetic joint infection.

Notes:Sensitivity and specificity are expressed as percentages (%).

### Multivariate linear regression analysis

To evaluate whether time-related variables confound the association between biomarkers and PJI status, we performed multivariate linear regression analyses with natural log-transformed biomarker levels as dependent variables, and group (PJI vs. AL), time from symptom onset, and time since implantation as independent predictors (**Table 7**).

**Table 7:** Multivariate linear regression analysis: association between biomarkers and time covariates in PJI versus AL.

Biomarker (Ln[Y + 1])	Group	Symptom duration (d)	Implantation time (mo)	R²	Adj. R²
	β	β	β		
Ln (CRP + 1)	1.8827 ^***^	-0.00008676	-0.001432	0.4913	0.4866
Ln (ESR + 1)	1.0783^***^	-0.00009325	-0.0002660	0.3269	0.3207
Ln (PLT + 1)	0.1274 ^**^	-0.000001221	-0.0007149^*^	0.0796	0.0711
Ln (PDW + 1)	-0.09107^***^	-0.000004729	-0.00001684	0.0494	0.0407
Ln (PLT/CRP)	-2.1720 ^***^	-0.0001273	0.0006500	0.4273	0.4220
Ln (PLT/ESR)	-0.8751 ^***^	0.00005121	-0.000008406	0.2501	0.2431
Ln (PDW/CRP)	-1.5383^***^	-0.00004894	0.001098	0.4355	0.4303

Abbreviations: d, days; mo, months. CRP, C-reactive protein; ESR, erythrocyte sedimentation rate; PLT, platelet count; PDW, platelet distribution width; PJI, periprosthetic joint infection; AL, aseptic loosening. All biomarkers were natural-log transformed (Ln[Y + 1]) prior to analysis.

Note: β coefficients represent the change in Ln(Y) per unit change in the predictor. Positive β for Group indicates higher levels in PJI; negative β for ratios indicates lower values in PJI. *P < 0.05, **P < 0.01, ***P < 0.001

All seven tested biomarkers remained significantly associated with PJI after adjustment for time-related variables(all *P* < 0.01). Notably, time from symptom onset showed no significant association with any of the tested biomarkers (all *P* > 0.05), indicating that symptom duration does not substantially influence the levels of these inflammatory markers. Time since implantation was only significantly associated with PLT levels (*P* = 0.0309), but had no significant effect on the other six biomarkers, including CRP, ESR, and all composite ratios.

The model fit was strongest for CRP (adjusted R² = 0.4866) and PDW/CRP (adjusted R² = 0.4303), suggesting that these markers are most strongly associated with PJI status after accounting for time-related covariates.

These regression results confirm that the observed differences in inflammatory biomarkers and composite ratios between PJI and AL groups are not confounded by symptom duration or implantation time.

### A pairwise comparison of ROC analysis on whether each test index predicts PJI

To further clarify whether the efficacy of PLT/CRP, PLT/ESR and PDW/CRP ratios in diagnosing PJI is superior to that of traditional serological indicators CRP and ESR, the method of Delong et al. (1988) was adopted to pairwise compare the ROC curves of each test indicator for predicting whether it is PJI. The diagnostic performance of combined parameters is summarized in **Table 8**. PDW/CRP achieved the highest AUC among all tested indicators, and there was no statistically significant difference between PDW/CRP and CRP (*Z* = 0.277, *P* = 0.7819). Similarly, no significant difference was observed between PLT/CRP and CRP (*Z* = 0.940, *P* = 0.3474). These results indicate that both platelet-CRP composite ratios possess diagnostic accuracy comparable to that of CRP alone.

**Table 8:** Pairwise comparisons of ROC analyses for laboratory markers in predicting PJI.

Indicator	ESR	PLT	PDW	PLT/CRP	PLT/ESR	PDW/CRP
CRP	*Z* = 2.610	*Z* = 7.201	Z=7.538	*Z* = 0.940	*Z* = 3.094	*Z* = 0.277
	*P* = 0.0091	*P* < 0.0001	*P <* 0.0001	*P* = 0.3474	*P* = 0.0020	*P* = 0.7819
ESR	_	*Z* = 5.970	*Z=*6.063	*Z* = 1.323	*Z* = 1.435	*Z* = 2.287
		*P* < 0.0001	*P* < 0.0001	*P=* 0.1859	*P* = 0.1512	*P* = 0.0222
PLT	_	_	Z=0.490	*Z* = 6.152	*Z* = 4.180	*Z* = 7.332
			*P* = 0.6239	*P* < 0.0001	*P* < 0.0001	*P* < 0.0001
PDW	_	_	_	*Z* = 6.617	*Z=*4.556	*Z* = 7.694
				*P* < 0.0001	*P* < 0.0001	*P<*0.0001
PLT/CRP	_	_	_	_	*Z=*2.870	*Z=*3.084
					*P =* 0.0041	*P* = 0.0020
PLT/ESR	_	_	_	_	_	*Z=*3.886
						*P =* 0.0001

PDW/CRP was significantly superior to ESR (*Z* = 2.287, *P* = 0.0222), PLT (*Z* = 7.332, *P* < 0.0001), PDW (*Z* = 7.694, *P* < 0.0001), and PLT/ESR (*Z* = 3.886, *P* = 0.0001). PLT/CRP also showed significantly higher diagnostic efficacy than single platelet markers (both *P* < 0.0001) and PLT/ESR (*P* = 0.0041).

Single platelet parameters (PLT and PDW) exhibited the lowest diagnostic performance among all tested markers, with no significant difference between them (*P* = 0.6239), and both were significantly inferior to all other indicators (all *P* < 0.0001).

## Discussion

The pathophysiological mechanism of PJI involves processes such as microbial colonization, biofilm formation, and activation of host immune responses. CRP, as an acute-phase reaction protein, significantly increases in the early stage of infection and is one of the most commonly used serum markers for diagnosing PJI (Osmon et al., 2013; Parvizi et al., 2018). However, its specificity is limited, and non-infectious inflammation (such as aseptic loosening and periprosthetic fractures) can also lead to elevated CRP (Sedlár et al., 2010; Chalmers et al., 2013). Platelets are not only important components of the hemostatic system but also key innate immune effector cells during bacterial infection. Human platelets express multiple functional Toll-like receptors (El Filaly et al., 2023), especially TLR4, which binds bacterial lipopolysaccharide and initiates downstream platelet activation, degranulation and pro-inflammatory signal transduction. Under infectious conditions, inflammatory factors stimulate thrombopoiesis by bone marrow megakaryocytes. Meanwhile, activated platelets release various inflammatory mediators and form a self-amplifying positive feedback loop. In severe infection such as PJI, continuous pathogen stimulation maintains persistent platelet activation. Activated platelets form microaggregates and are then cleared by circulating phagocytes, resulting in obvious platelet consumption and altered platelet volume heterogeneity (Setarehaseman et al., 2025). This immune-mediated consumption accounts for the obvious fluctuations in platelet counts and platelet distribution width (PDW) in patients with active PJI, and further improves the stability of platelet-to-CRP ratios for differentiating septic loosening from aseptic inflammation.

In clinical practice, a single platelet and its related indicators have limited value in diagnosing PJI infection because they are influenced by multiple factors (such as drugs, underlying diseases, etc.). However, CRP and ESR may show false negatives in the early postoperative period or in chronic low-virulence infections (Johnson et al., 2011). Our study found that platelet-derived composite ratios, particularly PDW/CRP, achieved diagnostic performance comparable to CRP, with the additional advantage of more balanced sensitivity and specificity. These ratios may help reduce false positive or false negative results caused by non-infectious factors when used as adjunctive tools alongside conventional markers. Therefore, the PLT/CRP and PDW/CRP ratio indicators proposed in this study may provide a simple, low-cost adjunctive diagnostic option. They do not require additional expensive tests and can be calculated solely based on routine blood tests and biochemical results. They may be particularly suitable for use in primary hospitals or as auxiliary indicators for preoperative screening, potentially assisting in early identification of suspected PJI cases.

Subgroup analyses in our study further support the stability of these composite ratios. No significant difference was observed in diagnostic performance between hip and knee joints for any marker, suggesting that joint location does not substantially influence the diagnostic value of these ratios. Notably, PDW/CRP demonstrated significantly higher AUC in acute PJI than in chronic PJI (*P* = 0.04071), indicating its potential advantage in identifying early-stage infections. Furthermore, subgroup analysis stratified by causative pathogen showed that PDW/CRP and PLT/CRP maintained stable and favorable diagnostic performance across both Staphylococcus aureus and coagulase-negative staphylococci (CoNS) infections, with no significant difference in AUC between the two pathogen types (all *P* > 0.05). In contrast, single PLT exhibited significantly lower diagnostic accuracy in S. aureus PJI (AUC = 0.5147) compared with CoNS PJI (AUC = 0.7356, *P* = 0.0201). The composite ratios effectively compensated for this instability of isolated platelet markers across different pathogen types, further demonstrating their robustness as auxiliary diagnostic indicators. Multivariate regression analyses confirmed that after adjusting for symptom duration and time since implantation, PJI status remained independently associated with all tested biomarkers, further suggesting that the observed differences are not confounded by time-related factors.

Xu (Xu et al., 2020)et al. reported that when platelet count (PLT) is used to diagnose periprosthetic joint infection (PJI), the area under the ROC curve is smaller than that of indicators such as ESR and CRP, which is consistent with the results of this study. The potential advantages of PLT/CRP and PDW/CRP lie in that they simultaneously reflect the inflammatory state and immune response of the body. In PJI, although both CRP and platelets may increase while PDW may decrease, the ratio of PLT/CRP and PDW/CRP can amplify abnormal signals under infection conditions. When the infection is severe, CRP rises sharply, while platelets may relatively decrease due to consumption or inhibition. Insufficient platelet production, together with uniform volume and reduced PDW, contributes to a significant reduction in the PLT/CRP and PDW/CRP ratios. This change is more specific than simply looking at the absolute values of CRP, PLT, or PDW.

It should be acknowledged that the diagnostic performance of platelet-CRP ratios is largely driven by the strong CRP signal, and platelet parameters contribute relatively modest additional diagnostic information in terms of overall AUC. However, the composite ratios offer advantages in terms of balanced sensitivity and specificity as well as improved robustness across different clinical subgroups, which supports their role as auxiliary screening tools rather than standalone diagnostic markers.

## Conclusion

This retrospective case-control study evaluated platelet-CRP ratios for PJI diagnosis after hip and knee arthroplasty. All biomarkers differed significantly between PJI and AL groups (all *P* < 0.0001). PDW/CRP achieved the highest AUC (0.9047) with well-balanced sensitivity and specificity (both ~85%), comparable to CRP (AUC = 0.9002) and superior to single platelet parameters. The ratios showed stable performance across joint types and pathogen species, with PDW/CRP performing better in acute PJI. After adjusting for time covariates, PJI status remained independently associated with all biomarkers.

Several limitations of this study should be acknowledged. This was a single-center retrospective case-control study with a non-consecutive sampling design, which may introduce spectrum bias and limit the generalizability of our findings. The relatively small sample size, particularly for subgroup analyses, may restrict statistical power. Another limitation is the imbalanced distribution of hip and knee arthroplasties between the PJI and AL groups. Although subgroup analyses showed comparable diagnostic performance across joint types, we cannot fully exclude the possibility that this imbalance may influence the primary combined analysis results. Future studies with matched joint distributions would help clarify this issue. Additionally, the optimal cut-off values derived from this study require external validation.

In conclusion, PLT/CRP and PDW/CRP ratios may serve as low-cost adjunctive indicators for the clinical diagnosis of PJI, with PDW/CRP exhibiting particularly well-balanced sensitivity and specificity. These ratios maintain stable performance across different joint types, pathogen types, and disease stages, and are especially useful for auxiliary judgment in suspected borderline or acute PJI cases when combined with routine inflammatory markers such as CRP. Large-scale prospective multicenter studies are warranted to further validate the clinical utility of these composite ratios in unselected arthroplasty populations.

## Data Availability

The original contributions presented in the study are included in the article/supplementary material. Further inquiries can be directed to the corresponding author.
